# Tuning Gelation of Insect Proteins: Effect of Ionic Strength on *Acheta domesticus* Protein Extracts

**DOI:** 10.3390/gels11120937

**Published:** 2025-11-21

**Authors:** Rossella Francesca Lanza, Eleonora Muccio, Francesca Malvano, Morteza Darvishikolour, Francesco Marra, Osvaldo H. Campanella, Donatella Albanese

**Affiliations:** 1 Department of Industrial Engineering, University of Salerno, Via Giovanni Paolo II, 84084 Fisciano, SA, Italy; rlanza@unisa.it (R.F.L.); emuccio@unisa.it (E.M.); fmalvano@unisa.it (F.M.); kasradarvishi22@gmail.com (M.D.); fmarra@unisa.it (F.M.); 2 Department of Agricultural, Forest and Food Sciences, University of Turin, Via Verdi 8, 10124 Turin, TO, Italy; 3 Department of Food Science and Technology, The Ohio State University, 2015 Fyffe Rd., Columbus, OH 43210, USA; campanella.20@osu.edu

**Keywords:** gels, protein gelation, ionic strength, rheology, techno-functional properties, *Acheta domesticus* proteins, sustainable proteins

## Abstract

Protein gelation is a key mechanism for structuring food systems, as it determines texture, water retention, and overall product stability. Therefore, understanding how processing factors influence gelation is critical for designing functional protein-based matrices. This study investigates the effect of ionic strength on the gelation and techno-functional properties of *Acheta domesticus* (house cricket) protein extract. Gels were prepared with increasing NaCl concentrations (0–0.5 M) and characterized by rheological analysis. Additionally, solubility, emulsifying properties and water/oil holding capacity of the protein extract were assessed. Small-amplitude oscillatory shear tests revealed that G′ increased from ~150 Pa at 0 M to over 1300 Pa at 0.5 M, indicating salt-induced network reinforcement. The loss factor (tan *δ*) reached its minimum (0.19) at high ionic strength, reflecting the formation of stronger, more elastic protein networks. These rheological trends aligned with the techno-functional responses: solubility peaked at 79.5% at 0.1 M NaCl before decreasing at higher salt levels, while emulsifying activity reached a maximum of 59.1 m^2^ g^−1^ at 0.3 M before dropping sharply. Collectively, these findings highlight ionic strength as a tunable parameter linking molecular interactions to bulk viscoelasticity and textural attributes. By adjusting salt concentration, elasticity, hydration, and interfacial stability can be strategically modulated, enabling the rational design of insect-based food gels for different applications, from emulsified systems to fibrous meat analogs.

## 1. Introduction

The global shift toward sustainable protein sources is reshaping food production systems, driven by the need to reduce environmental impacts while meeting the nutritional demands of a growing population [1,2]. Among these alternatives, insect proteins have gained attention for their favorable amino acid profile, digestibility, and resource efficiency compared to conventional livestock [3,4,5]. Among edible insects, *Acheta domesticus* (house cricket) is considered one of the most suitable species for food applications [6]. Although precise global production figures for crickets are not publicly available, recent reports provide insight into its industrial relevance. In the United States, the ten largest producers collectively yield over 1300 tonnes of live house crickets per year [7]. In Europe, a case-study facility in the United Kingdom reports an annual output of approximately 19 tonnes of crickets for the pet-food market [8]. Moreover, experimental farming trials report efficiencies of approximately 7.3 kg m^−2^ of lives crickets per production cycle [9]. These examples demonstrate that crickets is already farmed at an industrial scale in several regions, although the absence of harmonized reporting systems still limits the availability of consolidated global figures. Nevertheless, the commercial insect-farming sector overall is projected to exceed 3 million tonnes annually by 2030, with crickets representing a significant share [10].

As insect farming expands, the transition from raw biomass to functional protein ingredients becomes critical for integrating these materials into modern food systems. Beyond their nutritional potential, the successful utilization of insect proteins into structured foods depends largely on their techno-functional and gelation properties, which determine texture, stability, and, ultimately, consumer acceptance. Achieving these properties requires efficient and scalable protein extraction processes, able to preserve structural integrity while maximizing yield.

Commercial production of insect protein ingredients typically relies on mechanical, solvent-based, or alkaline extraction processes, each with specific advantages and limitations. The most widespread industrial approach is alkaline solubilization followed by isoelectric precipitation, commonly applied to crickets and other edible insects [4,11,12,13]. This method ensures high protein yield and good functional integrity, producing extracts with protein contents exceeding 80% and broad techno-functional versatility. However, it requires careful pH control to prevent irreversible denaturation and generates wastewater streams requiring neutralization, limiting its environmental sustainability. Alternative large-scale methods include mechanical pressing or wet fractionation, which avoid chemical reagents and preserve native protein structures, but usually result in lower purity and partial retention of lipids and chitin residues, affecting solubility and color [14]. Enzymatic or sonication-assisted extraction can enhance recovery efficiency and functionality [14,15]. However, these technologies are cost-intensive and less standardized for commercial throughput, which may restrict their adoption by small and medium producers. Overall, current industrial processing of insect proteins represents a balance between yield, functionality, and environmental impact; among these, the alkaline–isoelectric route remains the most scalable and widely adopted for producing food-grade cricket protein extracts.

Once extracted, the functional performance of these proteins during structuring processes such as gelation becomes a decisive factor for their practical application in food design. Protein gelation is a fundamental process in food structuring. Upon heat treatment or environmental changes, proteins unfold and associate into three-dimensional networks that govern water retention, elasticity, and rheological performance [16]. Such properties are particularly relevant in the development of meat analogs, where gels provide the cohesive texture and firmness needed to mimic muscle tissue [17]. In plant and dairy proteins, extensive work has shown that gelation and related functional attributes can be modulated by pH, thermal treatment, and ionic strength [18,19,20].

Particularly, ionic strength plays a pivotal role in controlling protein–protein interactions. By altering electrostatic forces and hydration layers, salts influence solubility, aggregation, and the balance between elasticity and brittleness in gels. Studies on soy, whey, and pea proteins have demonstrated that moderate salt concentrations enhance gel strength and water-holding capacity, while excessive ionic strength promotes aggregation and destabilization [21,22,23]. The effectiveness of ionic strength in modulating protein functionality also depends on the surrounding physicochemical environment, including pH, temperature, net surface charge, and the degree of structural unfolding induced during extraction, all of which shape protein–water and protein–protein interactions [24,25]. These findings underscore the importance of ionic environments in tuning protein gel performance.

To date, most available studies have focused on basic functional properties such as solubility, emulsification, and foaming capacity of insect proteins [11,12], while little attention has been paid to how controllable physicochemical factors, such as ionic strength, can be exploited to design tailored gel structures. A systematic understanding of these relationships is essential not only to advance the fundamental science of insect protein gelation but also to enable their rational application in food structuring.

The present study aims to investigate the effect of ionic strength on the gelation and techno-functional properties of cricket protein extracts. Rheological characterization of the cricket protein extract-based gels at different concentration of NaCl was combined with measurements of water-holding capacity, solubility, and emulsifying properties of the protein extracts. Finally, this work contributes to a broader understanding of protein gelation and identifies ionic strength as a tunable parameter for designing sustainable protein gels with potential applications in food structuring.

## 2. Results and Discussion

### 2.1. Techno-Functional Properties of the Protein Extract

#### 2.1.1. Protein Solubility

Protein solubility depends on multiple molecular factors, including the intrinsic composition of the extract, protein surface charge, and the degree of unfolding induced during alkaline extraction. The solvent exposure of amino acid chains, particularly hydrophilic and hydrophobic domains, further influences solubility. Additionally, ionic strength modulates these interactions by altering electrostatic repulsion and hydration, which in turn govern protein–protein association and dispersion in aqueous environments [18,19,20,21].

**Figure 1:**
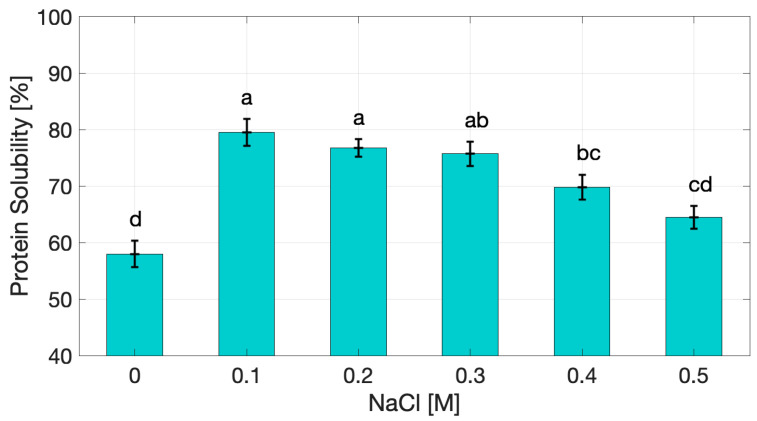
Protein solubility of cricket protein extract as a function of NaCl concentration. Data points followed by different letters (a–d) are significantly different (*p* < 0.05).

The solubility of cricket protein extracts (Figure 1) showed a clear dependence on ionic strength. Based on a total protein content of 85.7% (*w*/*w* dry extract) [26], at 0 M NaCl protein solubility was 58.0%, reflecting a good dispersion capacity of the extract in aqueous media. A statistically significant increase (*p* < 0.05) was observed at 0.1 M NaCl, where solubility peaked, while, beyond 0.2 M, solubility decreased progressively, reaching 64.42 ± 2.01% at 0.5 M (*p* < 0.05 compared to values at 0.1 M).

This observed trend is consistent with the classical “salting-in/salting-out” effect. At low ionic strength, NaCl ions shield electrostatic charges, reducing repulsion and allowing proteins to remain solvated. However, at higher concentrations, competition for water molecules leads to dehydration of protein surfaces, promoting hydrophobic interactions and aggregation [21,27].

Comparable solubility levels have been reported for other insect protein isolates, including *Gryllodes sigillatus*, *Acheta domesticus*, and *Hermetia illucens*, which typically exhibit solubility values between 50% and 65% at neutral pH [4,11,28]. While detailed investigations on the effect of ionic strength on insect proteins remain limited, similar salting-in and salting-out behavior has been extensively described for plant and animal globular proteins such as soy, pea, whey, and egg white isolates [29,30]. In particular, soy protein isolates typically show sharp decline above 0.3 M NaCl [31], while pea protein isolates exhibit 40–50% reduction under similar conditions [22]. These parallels suggest that cricket protein extract follows comparable physicochemical mechanisms, supporting its functional equivalence to conventional protein systems across different biological origins.

From a functional standpoint, solubility is a prerequisite for effective participation in network formation and interfacial activity. Proteins must be sufficiently dispersed to unfold, interact, and form structured gels upon heating or other treatments [18]. However, partial loss of solubility at higher ionic strength does not necessarily hinder gelation; instead, it can promote protein–protein interactions that lead to denser and more elastic networks. This dual effect highlights a functional trade-off between network strengthening and interfacial performance. Similar behavior has been reported for soy and whey protein isolates, where reduced solubility at high salt concentrations is associated with stronger gels but poorer emulsifying stability [32,33]. These results underscore the complex role of solubility in the functional behavior of insect protein extracts, influencing gel elasticity and interfacial properties in opposite ways depending on ionic strength.

Interestingly, although the trend mirrors that of soy and pea proteins, the absolute solubility values of cricket extracts remained relatively high at the uppermost value of investigated ionic strength (≥64% at 0.5 M NaCl), suggesting a certain stability of solubility under ionic stress. This may be linked to the heterogeneous composition of the insect extract, which contains both readily soluble globular proteins (e.g., enzymatic and storage proteins) and less soluble structural or cuticle-associated proteins. The coexistence of these fractions may broaden the functional window of the extract compared with purified isolates, maintaining dispersion even under elevated salt conditions [4,12]. The stability of solubility of the protein extracts may represent an advantage in food processing, where protein systems are often subjected to fluctuating ionic conditions.

#### 2.1.2. Water-Holding Capacity and Oil-Holding Capacity

**Figure 2:**
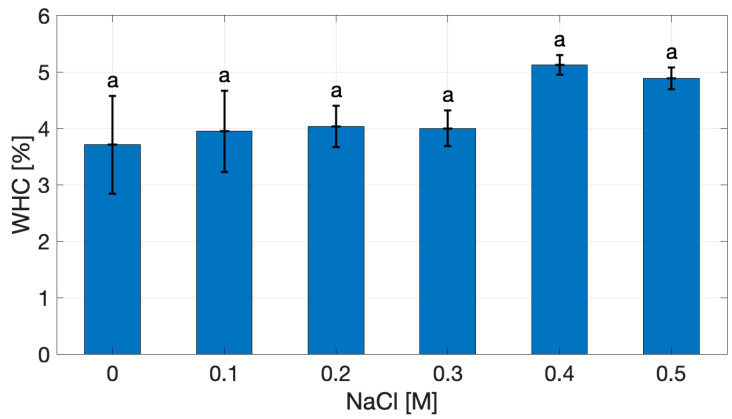
Water-Holding Capacity of cricket protein extract as a function of NaCl concentration. Same superscript letter represents non-significant differences (*p* > 0.05).

The Water-Holding Capacity (WHC) of cricket protein extracts was not affected by ionic strength, as shown in Figure 2. At 0 M NaCl, WHC is 3.71 ± 0.61% (*w*/*w* dry extract), reflecting the hydration ability of the extract. Across the investigated ionic strength range, WHC values showed no significant differences (*p* > 0.05), varying from 3.94% to 4.88%, with the highest mean value observed at 0.4 M NaCl. Although the differences were not significant, the upward trend suggests that moderate salt concentrations may enhance protein–protein interactions through electrostatic screening, promoting water entrapment within the protein matrix without causing extensive aggregation [19,20,21,22].

Comparable WHC values have been reported for protein isolates from other edible insects, such as *Acheta domesticus*, *Gryllodes sigillatus*, and *Tenebrio molitor*, typically ranging between 2.5 and 4.5 g water/g protein [11,12,34,35]. These values are also consistent with those of plant proteins such as soy and pea isolates (2.5–4.0 g/g) [27,29], whereas animal proteins like whey or egg-white isolates generally exhibit slightly higher WHC (3.0–4.5 g/g) [24,25].

The similarity between cricket protein extract and animal proteins suggests that alkaline extraction conditions preserved sufficient conformational flexibility and solvent-accessible polar domains, enabling effective hydration and water retention [12,25].

This observation is particularly relevant when considered alongside solubility behaviors: although solubility decreased at high ionic strength, WHC increased, suggesting that proteins excluded from the soluble fraction contributed effectively to matrix formation and water retention.

From a food structuring perspective, higher WHC at elevated salt levels suggests the potential to produce gels with improved juiciness and reduced syneresis, properties essential in applications such as meat analogs.

The Oil-Holding Capacity (OHC) of the dialyzed protein extract was 3.98 ± 0.03% (*w*/*w* dry extract). This high value indicates a strong lipophilic interaction capacity, likely related to the exposure of hydrophobic amino acid residues during extraction. Similar relationships between hydrophobic exposure and lipid binding capacity have been reported for plant and animal protein systems [24,25]. From a functional perspective, high OHC suggests that the extract can bind non-polar molecules such as lipids and volatile flavor compounds, which is advantageous for the development of stable and palatable food systems, including emulsions, dressings, and fat-rich matrices [19]. Similar OHC ranges (2.5–4.0 g oil/g protein) have been reported for *Acheta domesticus* and *Alphitobius diaperinus* protein isolates [4,13,35], and are comparable to those observed for soy protein concentrates (3–4 g/g) [36]. Minor differences may arise from residual lipid content, surface hydrophobicity, and the degree of unfolding induced by alkaline conditions [12,24,25].

Although OHC was not measured across varying ionic strengths, it remains an important complementary property to WHC. Whereas WHC governs gel hydration and elasticity, OHC represents the capacity of the extract to bind lipids, thereby contributing to improved sensory perception and formulation stability.

#### 2.1.3. Emulsifying Properties

**Figure 3:**
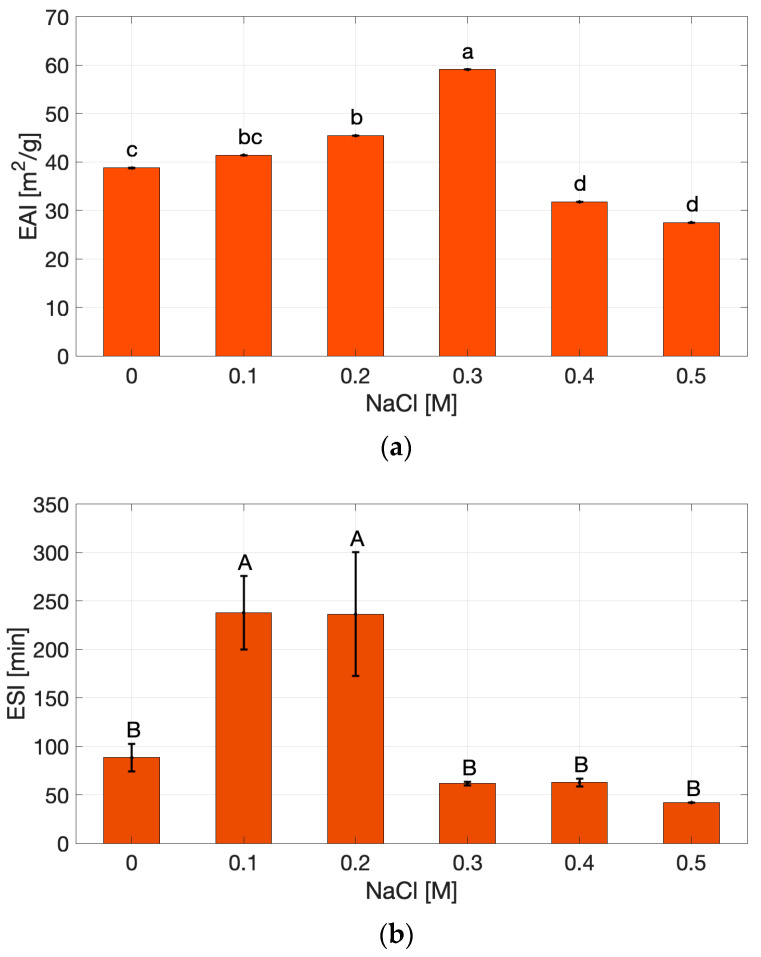
Emulsion Activity Index (**a**) and Emulsion Stability Index (**b**) of cricket protein extract under varying NaCl concentrations. Values marked with different letters (a–d and A–B) indicate statistically significant differences (*p* < 0.05).

The Emulsifying Activity Index (EAI, Figure 3a) and Emulsion Stability Index (ESI, Figure 3b) of cricket protein extracts showed different non-monotonic responses to the NaCl concentration.

The EAI progressively increased from 0 to 0.3 M NaCl, where the highest activity was recorded, and then declined at higher concentrations, reaching values comparable to the salt-free extract. In contrast the ESI reached its highest value at 0.1 M NaCl (*p* < 0.05) and then declined sharply beyond 0.2 M, showing the lowest stability at 0.5 M. The increase in EAI up to 0.3 M may be attributed to partial charge screening that facilitates protein adsorption and interfacial packing, as widely reported for globular protein systems [33,37,38]. At moderate ionic strength, salt ions reduce electrostatic repulsion between protein molecules, enabling closer packing at the oil–water interface and improving interfacial coverage.

However, the simultaneous drop in ESI at high salt concentrations suggests that, despite faster interfacial coverage, the interfacial films formed are more susceptible to flocculation/coalescence due to strong screening of electrostatic repulsions between droplets—an effect observed in protein-stabilized emulsions at elevated ionic strength [38]. This behavior can be explained by excessive charge screening, which suppresses electrostatic stabilization between emulsion droplets, which is a commonly observed effect in protein-stabilized emulsions at elevated ionic strength [33,38]. At 0.5 M NaCl, both EAI and ESI were low, consistent with bulk aggregation and reduced solubility, which limit the number of mobile, interfacially active molecules [31]. These trends are consistent with the solubility behavior, where higher ionic strength promoted aggregation and reduced protein dispersion. Lower solubility consequently diminishes the number of surface-active molecules available to migrate and stabilize oil droplets. Conversely, at low to moderate salt concentrations, where solubility remains high, proteins are sufficiently unfolded and flexible to efficiently adsorb at the interface, explaining the higher EAI and ESI values observed in this range. The relatively high OHC of the extract further supports its intrinsic emulsifying potential, since exposed hydrophobic residues favor anchoring at the oil–water interface. Together, these results suggest that ionic strength modulates emulsifying behavior through its simultaneous influence on solubility and hydrophobic interactions, balancing dispersion and aggregation within the system.

EAI and ESI results are compatible with reports on soy proteins under NaCl, where moderate salt can improve interfacial activity, but excessive ionic strength induces aggregation and destabilization [31], and align with observations on insect protein extracts, whose emulsifying performance is sensitive to ionic environment and dispersion state [4].

### 2.2. Rheological Properties of Protein Gels

**Figure 4:**
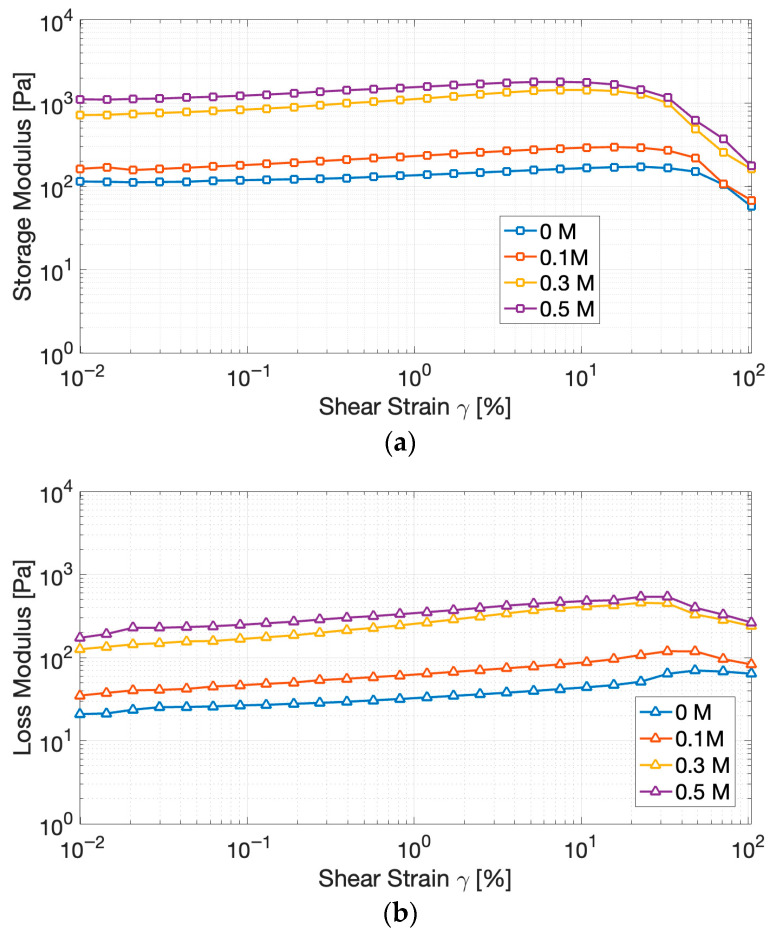
Evolution of the storage modulus (G′) (**a**) and of the loss modulus (G″) (**b**) of cricket protein gels at varying NaCl concentrations during amplitude sweep tests.

To evaluate the effect of ionic strength on the viscoelastic behavior of the gels, amplitude sweep tests were carried out. The results (Figure 4) revealed an elastic-dominant profile (G′ > G″), confirming the establishment of three-dimensional protein networks, capable of storing deformation energy rather than dissipating it through flow [39].

The Linear Viscoelastic Region (LVR) decreased with increasing salt concentration, indicating that the gels became less tolerant to deformation before structural breakdown. Simultaneously, the storage modulus (G′) rose consistently with NaCl, from less than 200 Pa at 0 M NaCl to more than 1300 Pa at 0.5 M, reflecting denser and more cross-linked protein assemblies.

These changes denote that moderate to high ionic strength enhances protein–protein interactions by screening repulsive charges and favoring hydrophobic associations. However, yield strain shifted downward, reflecting a trade-off: stronger gels formed, but they became more brittle and prone to fracture under stress. Similar outcomes have been observed in pea and soy gels [40,41], where the addition of salts increases cross-linking and rigidity at the expense of plasticity.

Interestingly, a distinctive and somewhat atypical observation derived from the amplitude sweep tests is the initial ascending trend in both moduli within what is conventionally defined as the LVR. In ideal gel systems, the LVR is rigorously defined by a plateau region where G′ and G″ maintain constant values, indicating that the applied strain is insufficient to induce disruption of the network structural integrity [42]. In the present case, a perfectly linear region could not be clearly identified; nevertheless, the strain interval preceding the decrease in G′ can be reasonably considered an apparent LVR, since the variation of G′ remained within approximately 10% of its initial value, which is an operational criterion commonly applied in non-ideal gel systems [42,43]. Overall, we attribute the observed increase in the moduli to either slow gel maturation during testing or to minor strain-induced microstructural rearrangements that transiently enhance network connectivity. Similar low-strain stiffening or alignment phenomena have been reported in whey protein systems, where a mild strain-hardening response within the nominally linear region was attributed to microstructural rearrangements and progressive formation of junction zones that temporarily increase network connectivity and stiffness [43]. Additional analyses would help discriminate between time-dependent aging and genuine strain-induced stiffening.

**Figure 5:**
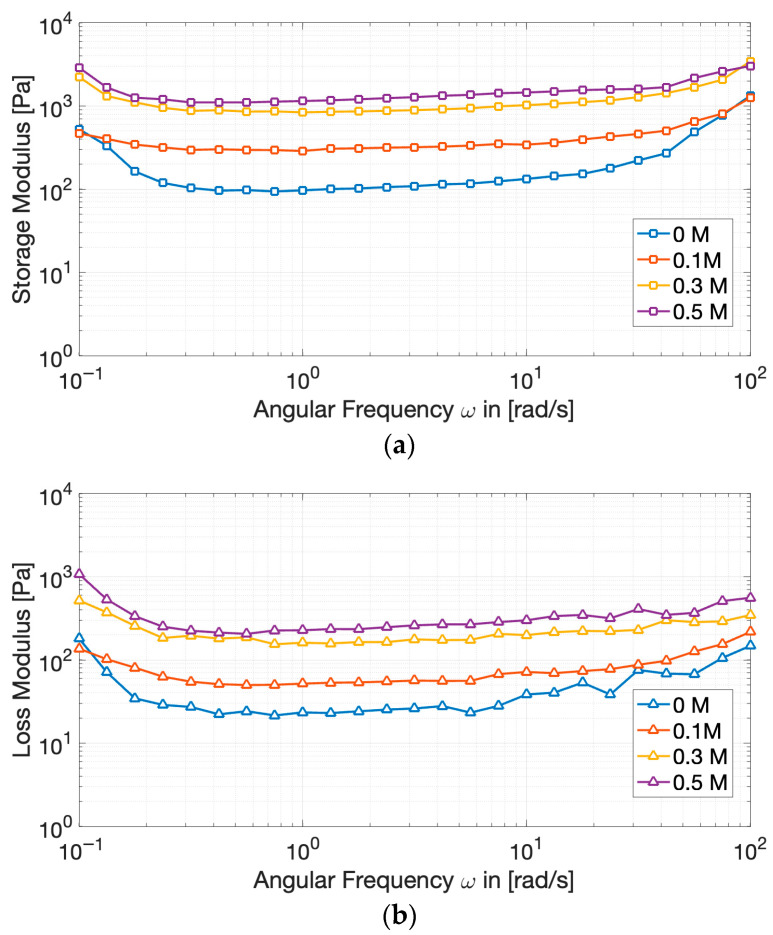
Evolution of the storage modulus (G′) (**a**) and of the loss modulus (G″) (**b**) of cricket protein gels at varying NaCl concentrations during frequency sweep tests.

Frequency sweeps (Figure 5) further underline the solid-like nature of the gels across the 0.1–100 Hz range: the relatively limited dependence of both G′ and G″ on angular frequency, in fact, reinforces the conclusion that stable, predominantly elastic, cross-linked networks were successfully formed [44].

This behavior is indeed characteristic of gel-like structure, wherein the intrinsic relaxation times of the network significantly exceed the inverse of the applied oscillation frequency.

Increasing NaCl concentration causes G′ to rise quickly, whereas G″ increases only slightly, resulting in lower tan *δ* values at higher salt levels. The lowest value of tan *δ*, registered at 0.3 M NaCl (Table 1), corresponds to the most elastic and energy-storing gels. Such behavior suggests that the presence of salt promotes a more interconnected and less dissipative network, consistent with the “salting-in” effect on partially aggregated proteins that reorganize into continuous structures during heating. At 0.5 M NaCl, tan *δ* remained statistically comparable to that at 0.3 M (*p* > 0.05), suggesting that gel elasticity reached a plateau. This suggests that further ionic screening no longer strengthens the network measurably; instead, excessive salt may begin to promote slight phase separation or weaker junction zones at the microstructural level [21,23,37]. Nevertheless, the overall elastic dominance of the gels was maintained, as tan *δ* values remained significantly lower than the control (0 M).

To further quantify these rheological trends and better interpret the structural evolution of the gels, each sweep was fitted to a power-law model, according to Equations (6) and (7) within the linear, monotonic region of the log–log plots (Table 2).

This approach is widely adopted for characterizing protein and biopolymer gels under small-amplitude oscillatory shear, proximate scaling accurately captures the viscoelastic behavior [20,40,45,46,47]. This approach provides complementary insight into the mechanical strength and organization of the protein network beyond the qualitative trends of G′ and G″. The consistency indices k′ and k″, related to the magnitude of G′ and G″, respectively, increase markedly with salt concentration, confirming the progressive reinforcement of the gel matrix. The largest rise of both indices is observed between 0.1 and 0.3 M NaCl, indicating the formation of a stable, compact network capable of storing mechanical energy. The frequency-dependence exponents n′ and n″ quantify the sensitivity of the moduli to oscillation frequency: as NaCl increases, both n′ and n″ decreases, implying that both G′ and G″ become progressively less frequency-dependent. The strong increase in k′ and the corresponding decrease in n′ with NaCl concentration highlight the transition from soft, weakly connected aggregates to densely cross-linked, elastic structures. This trend is characteristic of well-developed gels in which structural relaxation is limited—a pattern also reported for plant proteins under similar ionic conditions [40,41]. Moreover, the combined reduction in n values and tan *δ* thus reflects a transition from weak, entangled networks toward elastic, solid-like matrices, reinforcing the mechanistic role of ionic screening in promoting junction zone formation and stabilizing the gel network. Hence, the power law modeling framework validates the observed experimental evolution of G′ and tan *δ*, confirming that ionic strength drives a shift from viscous-dominated to elasticity-dominated behavior.

**Figure 6:**
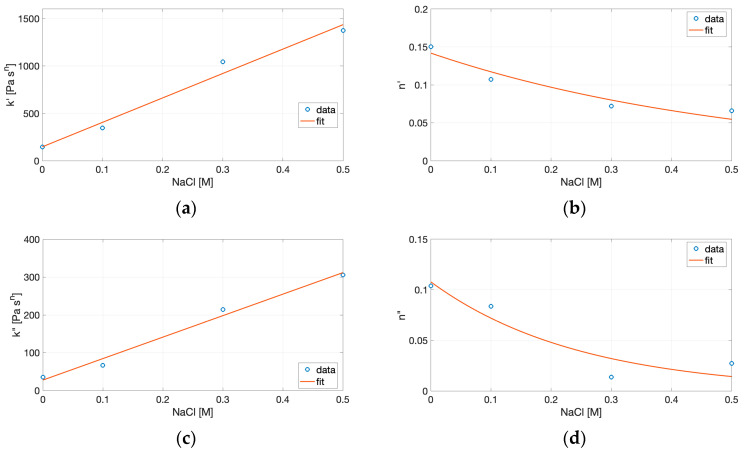
Variation in the power-law parameters with NaCl concentration: (**a**) consistency index k′, (**b**) frequency-dependence exponent n′, (**c**) consistency index k″, and (**d**) frequency-dependence exponent n″. Symbols represent experimental data, and solid lines correspond to the fitted correlations.

To connect the power-law model with the compositional variable, the fitted parameters were empirically correlated with NaCl concentration. Linear regressions were applied to the consistency indices (k′, k″), while exponential functions were used for the frequency-dependence exponents (n′, n″), as described by Equations (8) and (9). The fitting coefficients derived from these models are summarized in Table 3, and the resulting trends of the power-law parameters as a function of NaCl concentration are illustrated in Figure 6.

Although purely descriptive, these relationships provide a functional map linking the rheological parameters to ionic conditions, allowing identification of the salt range associated with maximal stiffness (high k′) and minimal frequency dependence (low n′). Such empirical correlations are particularly useful in translating experimental rheological data into formulation strategies. By expressing k′ and n′ as functions of NaCl concentration, it becomes possible to predict the mechanical response of cricket protein gels under given ionic environments without performing a full frequency sweep. This approach is consistent with prior modeling of biopolymer networks, where power-law parameters have been shown to capture the degree of cross-linking, relaxation behavior, and energy dissipation in protein and polysaccharide gels [46,47].

Taken together, these empirical correlations can be viewed as a useful descriptive framework for understanding how ionic strength modulates gel stiffness and relaxation dynamics. However, the limited number of experimental points constrains their statistical robustness and prevents rigorous extrapolation. Extending the analysis to a broader ionic strength range and performing independent validation would be necessary to confirm the quantitative and predictive value of these relationships for protein gel systems.

From a molecular standpoint, this evolution, as captured by the fitted power-law parameters, can be attributed to electrostatic screening that facilitates close packing and hydrophobic clustering of partially unfolded protein molecules, leading to a greater number of effective junction zones. According to previous results, the increase in G′ and k′ parallels the trends in WHC, indicating that tighter matrices retain more water, while the decline in solubility confirms that aggregation rather than dispersion dominates the behavior at elevated ionic strength.

Conversely, the same compactness that strengthens elasticity reduces protein availability at the oil–water interface, impairing emulsifying properties. Thus, rheology bridges solubility, WHC, and emulsification: salt promotes aggregation, which enhances gel elasticity and hydration but diminishes interfacial stability. Such a balance is crucial in food applications where different textures and functionalities are targeted.

In practical terms, understanding how ionic strength modulates gelation in cricket proteins is relevant for food manufacturers seeking to replace animal proteins or optimize texture in sustainable products such as meat analogs and protein-enriched snacks [1,2,3,6]. These results also contribute to the broader scientific understanding of protein gelation by linking ionic interactions to the formation of insect protein networks [18,19,20,22]. The study therefore adds new insights into the functional behavior of insect proteins and offers practical guidance for food manufacturers and policymakers aiming to promote sustainable protein sources and consumer acceptance of insect-based foods [1,2,5,6].

## 3. Conclusions

This work demonstrated that ionic strength plays a pivotal role in tuning both the techno-functional properties and the gelation behavior of *Acheta domesticus* (house crickets) protein extracts. At moderate salt concentrations, these interactions led to the formation of elastic, water-rich gels, whereas excessive ionic strength promoted extensive aggregation resulting in stronger but less functional matrices. Rheological analyses and power-law modeling consistently confirmed the progressive transition from weakly connected aggregates to dense, solid-like networks, providing quantitative evidence of the structural reinforcement induced by salt addition.

These findings establish ionic strength as a tunable parameter linking molecular interactions to macroscopic functionality. By adjusting salt concentration, it is possible to strategically control elasticity, water retention, and interfacial stability, thus tailoring the texture and functional performance of insect-based protein gels. From a technological standpoint, this tunability opens opportunities for developing formulations with desired textures, ranging from soft emulsified systems to firm, cohesive gels, while potentially reducing the need for added hydrocolloids in line with clean-label trends.

Further studies are required to improve process reproducibility, assess storage stability, and validate rheological models on a larger dataset to enable predictive formulation tools. Evaluating performance in complex food matrices and under industrial processing will clarify the applicability of salt-responsive cricket protein gels in real manufacturing environments. From an industrial perspective, the salt-dependent structuring behavior of cricket proteins offers opportunities to tailor texture and water retention in meat analogs, high-protein snacks, and emulsified products, potentially reducing hydrocolloid use and supporting clean-label formulations.

## 4. Materials and Methods

### 4.1. Materials

The cricket flour was purchased from Small Giants S.r.l. (Milan, Italy). The nutritional composition of the flour, as reported by the manufacturer, is reported in Table 4. The Pierce™ Bradford Protein Assay reagent was purchased from Thermo Fisher Scientific Inc. (Waltham, MA, USA) The Slide–A–Lyzer dialysis flasks were purchased from Thermo Fisher Scientific Inc. (Waltham, MA, USA). All other chemicals were of analytical grade and purchased from Merck (Darmstadt, Germany). The palm oil was purchased from Euronut s.p.a (Avellino, Italy).

### 4.2. Protein Extraction

Cricket flour was first defatted [13] by solvent extraction with n-hexane (1:5, *w*/*v*) under continuous stirring for 3 h at room temperature, followed by centrifugation (SL 8 Small Benchtop, Thermo Fisher Scientific, Waltham, MA, USA) at 3100× *g* for 10 min and overnight air-drying of the pellet under a fume hood to remove residual solvent.

Subsequently, protein extraction was carried out by alkaline solubilization coupled with isoelectric precipitation [14]: the defatted flour was suspended in 0.25 M NaOH (1:15, *w*/*v*), stirred for 2 h at 40 °C, and centrifuged at 3500× *g* for 20 min; a second extraction was repeated on the pellet under the same condition and pooled supernatants were adjusted to pH 4.4 with 1 M HCl to induce precipitation. The protein extract was collected by centrifugation (2500× *g*, 20 min), neutralized to pH 7.0 and freeze-dried. According to our previous study, the protein content of the extract was close to 86% and the protein extraction yield, calculated as the percentage of total protein recovered in the extract relative to the amount of protein originally present in the defatted cricket flour, was approximately 61% [26]. To remove salts and low-molecular-weight compounds, protein extract suspensions (12% *w/v* in distilled water) were dialyzed in 3.5 kDa MWCO cassettes (Slide–A–Lyzer dialysis, Thermofisher, Waltham, MA, USA) against distilled water under gentle stirring for 24 h [48]. Dialyzed suspensions were recovered, freeze-dried, and used for subsequent analyses.

### 4.3. Protein Solubility

Protein solubility was determined according to a colorimetric method based on the Bradford assay [49], with modifications. Cricket protein extract (0.1% *w*/*v*) was dispersed in Phosphate-Buffer Solutions (PBS, 0.1 M, pH 7) with NaCl concentration ranging from 0.1 to 0.5 M. A dispersion of the extract in PBS without added salt was used as control. Samples were stirred for 60 min at room temperature and centrifuged at 3200× *g* for 20 min. Supernatants (50 μL) were mixed with 1.5 mL of Pierce™ Bradford Protein Assay reagent, incubated for 10 min, and absorbance was measured at 595 nm using a UV-6300PC spectrophotometer (VWR International, Leuven, Belgium). Blanks were prepared with the corresponding buffer and assay reagent. Protein concentrations were quantified against a calibration curve prepared with bovine serum albumin as standard.

Solubility was calculated as the percentage of soluble protein (P_Soluble_) relative to the total protein content (P_Total_), according to Equation (1):

(1) $$Solubility\left(\%\right)=\frac{P_{Soluble}}{P_{Total}}\;100$$

Total protein content was determined by the Kjeldahl method [50], using 6.25 as a nitrogen-to-protein conversion factor.

### 4.4. Water-Holding Capacity and Oil-Holding Capacity

The Water-Holding Capacity (WHC) and Oil-Holding Capacity (OHC) of the samples were evaluated following gravimetric method [51], with minor modifications. Briefly, 0.1 g of sample was suspended in PBS at different concentration of NaCl (0 to 0.5 M). PBS/palm oil was slowly added to the centrifugal tube until the sample was covered and then, tubes were vortexed for 1 min (REAX 2000, Heidolph GmbH, Schwabach, Germany), left to stand at room temperature for 30 min, and centrifuged at 3200× *g* for 15 min at 20 °C. The supernatant was carefully decanted, and the retained PBS/oil was quantified gravimetrically.

WHC and OHC were expressed as the percentage of water or oil retained by the extract relative to the total mass of the sample on dry basis, according to the following equations:

(2) $$WHC\left(\%\right)=\frac{m_{2}-m_{1}}{m_{0}}\;100$$

(3) $$OHC\left(\%\right)=\frac{m_{2}-m_{1}}{m_{0}}\;100$$

where $m_{0}$ is the mass (g) of the protein extract (dry basis), $m_{1}$ is the initial weight of the centrifuge tube with the sample, and $m_{2}$ is the final weight of the tube after supernatant removal.

### 4.5. Emulsifying Properties

The evaluation of the emulsifying activity index (EAI) and emulsion stability index (ESI) was conducted through turbidimetric method [15]. Briefly, a 0.1% *w/v* protein extract solution was prepared in PBS (0.1 M, pH 7.0) and different NaCl concentrations, ranging from 0 to 0.5 M. Emulsions were prepared by homogenizing the protein solution and palm oil at a 1:4 (*v*/*v*) ratio using an Ultra-Turrax T25 homogenizer (IKA, Staufen, Germany) at 10,000 rpm for 1 min. Aliquots (50 μL) of each emulsion were collected immediately after homogenization and after 10 min, diluted in 5 mL of 0.1% *w/v* SDS solution, and absorbances were measured at 500 nm (UV–Visible spectrophotometer, VWR International, Radnor, PA, USA), with 0.1% SDS solution as blank control. EAI (m^2^/g) and ESI (min) were calculated using the following equations:

(4) $$EAI\left(\frac{m^{2}}{g}\right)=\frac{2.303\;2A_{o}DF}{C\varphi \theta 10^{4}}$$

(5) $$ESI\left(min\right)=\frac{A_{0}}{A_{0}-A_{t}}∆t$$

where A_0_ and A_10_ are the absorbances of the diluted emulsions at 0 min and 10 min, respectively, DF is the dilution factor (100), C is the concentration of protein (g/mL) before emulsification, φ is the proportion of the oil phase (0.25), and θ is the optical path (1 cm), 10^4^ is a unit conversion factor to express EAI as m^2^/g and Δt is equal to 10 min.

### 4.6. Gel Preparation

Protein gels were prepared from cricket protein extract (15% *w/v*) in PBS containing different NaCl concentrations (0.1, 0.3 and 0.5 M), following a thermal gelation method [36], with modifications. Solutions were heated in a water bath (WB-M5, Falc, Treviglio, Italy) to 90 °C to induce gelation, held at this temperature for 15 min, cooled at room temperature for 10 min, and subsequently stored at 4 °C for 20 min to complete gel formation. A control gel was prepared using PBS without NaCl addition under the same conditions.

### 4.7. Rheological Characterization

The viscoelastic properties of the gels were evaluated using a rotational rheometer (MCR 102e, Anton Paar GmbH, Graz, Austria) equipped with a Peltier temperature device (P-PTD200/56) and plate–plate geometry (25 mm diameter, PP25 system). Immediately after preparation, gel samples were carefully loaded onto the rheometer, and the upper plate was adjusted to a final gap of 2 mm [40]. All rheological measurements were conducted at 20 °C

#### 4.7.1. Amplitude Sweep

Amplitude sweep tests were conducted at a fixed frequency of 0.1 Hz to determine the linear viscoelastic region (LVR), with strain ranging from 0.01% to 100%. The RheoCompass^®^ (version 1.32) software (Anton Paar GmbH, Graz, Austria) provided the storage modulus (G′, Pa) and loss modulus (G″, Pa), which reflect the elastic and viscous behavior of the gels, respectively. The yield strain was defined as the strain point where the storage modulus (G′) began to decline by more than 5%, indicating the onset of structural breakdown [41].

#### 4.7.2. Frequency Sweep

Frequency sweep tests were performed within the LVR over a frequency range of 0.1–100 Hz. The storage modulus (G′, Pa) and loss modulus (G″, Pa) were recorded and the loss factor (tan *δ* = G″/G′) was also provided by the software as indication of the balance between viscous and elastic contributions to the gel network. The frequency sweep data were fitted using a power law model in order to describe the dependence of the storage modulus (G′) and the loss modulus (G″) on the angular frequency (ω), as expressed in Equations (6) and (7) [52]:

(6) $$G^{\prime }\left(Pa\right)=k^{\prime }{(\omega )\;\;}^{n^{\prime }}$$

(7) $$G^{\prime \prime }(Pa)=k^{\prime \prime }{(\omega )\;\;}^{n^{\prime \prime }}$$

where k′ and k″ are the consistency indices associated with G′ and G″, respectively, and n′ and n″ represent the frequency-dependence exponents.

To integrate the power-law modeling with the compositional variable, the parameters were empirically correlated with NaCl concentration using linear and exponential fits for the consistency indices and the exponents, respectively:

(8) k=a x+b

(9) $$n=c\;e^{dx}$$

where a, b, c and d are fitting coefficients, and x represents the NaCl concentration.

### 4.8. Statistical Analysis

All measurements were performed in triplicate, and results are expressed as mean ± standard deviation (SD). One-way analysis of variance (ANOVA) was applied to evaluate overall differences among treatments. When ANOVA indicated significance (*p* < 0.05), Tukey’s honest significant difference (HSD) post hoc test was used to perform pairwise comparisons between groups. Statistical analyses were conducted using JMP statistical (version 18) software (SAS Institute. Inc. Cary, NC, USA).

## Figures and Tables

**Table 1:** Loss factor values for cricket protein extract gels at different NaCl concentrations.

NaCl [M]	tan *δ*
0	0.31 ± 0.14 ^A^
0.1	0.28 ± 0.06 ^AB^
0.3	0.19 ± 0.04 ^C^
0.5	0.24 ± 0.08 ^BC^

Values followed by different letters (A–C) are significantly different (*p* < 0.05).

**Table 2:** Parameters of the power law model for cricket protein extract gels at different NaCl concentrations.

NaCl [M]	k′ [Pa·s^n^]	k″ [Pa·s^n^]	n′	n″
0	145.54	35.43	0.15	0.1
0.1	343.43	66.91	0.11	0.08
0.3	1042.90	214.50	0.07	0.01
0.5	1372.10	305.61	0.06	0.03

**Table 3:** Fitting parameters of the empirical correlations between the power-law coefficients (k′, k″, n′, n″) and NaCl concentration.

Parameter	a	b	R^2^	c	d	R^2^
k′	2577.60	146.64	0.977	-	-	-
k″	568.12	27.78	0.986	-	-	-
n′	-	-	-	0.14	−1.91	0.826
n″	-	-	-	0.11	−4.05	0.775

**Table 4:** Nutritional composition of the cricket flour as stated by the manufacturer.

Nutrient (per 100 g)	Value [g]
Fat	11.60
Carbohydrates	4.60
Fiber	8.80
Protein	74.60
Salt	0.395

## Data Availability

The original contributions presented in this study are included in the article. Further inquiries can be directed to the corresponding author.

## Abbreviations

The following abbreviations are used in this manuscript:

EAI	Emulsifying Activity Index
ESI	Emulsion Stability Index
G′	Storage Modulus
G″	Loss Modulus
k′	Consistency index of the storage modulus in the power-law model
k″	Consistency index of the loss modulus in the power-law model
LVR	Linear Viscoelastic Region
MWCO	Molecular Weight Cut-Off
n′	Frequency-dependence exponent of the storage modulus in the power-law model
n″	Frequency-dependence exponent of the loss modulus in the power-law model
OHC	Oil-Holding Capacity
PBS	Phosphate-Buffer Solution
SDS	Sodium dodecyl sulfate
tan δ	Loss factor (ratio of loss modulus G″ to storage modulus G′)
WHC	Water-Holding Capacity
